# What counts as an embryo? The regulatory status of stem cell-based embryo models in Asia

**DOI:** 10.1038/s44319-026-00903-4

**Published:** 2026-08-19

**Authors:** Tsutomu Sawai, Shu Ishida, Chie Kobayashi, Yasuna Murase Ando, Daniel Fu-Chang Tsai, Ji Hyun Yang, Ilhak Lee, Tamra Lysaght, Mohammad Firdaus Bin Abdul Aziz, Julian Savulescu

**Affiliations:** 1 Graduate School of Humanities and Social Sciences, Hiroshima University, Higashi-Hiroshima, Japan; 2 Uehiro Division for Applied Ethics, Graduate School of Humanities and Social Sciences, Hiroshima University, Higashi-Hiroshima, Japan; 3 Centre for Biomedical Ethics, Yong Loo Lin School of Medicine, National University of Singapore, Singapore, Singapore; 4 Faculty of Science and Technology, Keio University, Yokohama, Japan; 5 Graduate Institute of Medical Education and Bioethics, National Taiwan University College of Medicine, Taipei, Taiwan; 6 Department of Medical Humanities, Chungnam National University College of Medicine, Daejeon, Korea; 7 Asian Institute for Bioethics and Health Law, Yonsei University, Seoul, Korea; 8 Division of Medical Law and Ethics, Department of Medical Humanities and Social Sciences, College of Medicine, Yonsei University, Seoul, Korea; 9 Sydney Health Ethics, Sydney School of Public Health, The University of Sydney, Faculty of Medicine and Health, Sydney, NSW Australia; 10 Faculty of Law, Universiti Malaya, Kuala Lumpur, Malaysia; 11 Uehiro Oxford Institute, University of Oxford, Oxford, UK

**Keywords:** Development, Economics, Law & Politics, Stem Cells & Regenerative Medicine

## Abstract

This paper examines legal frameworks and ongoing debates concerning research with human stem cell–based embryo models (SCBEMs) in China, Japan, Malaysia, Singapore, South Korea, and Taiwan. Given their great potential for research and their ethical implications, insights from these legislations could help to clarify and formulate global consensus on the regulation of SCBEM research.

**Figure:**
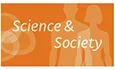

In recent years, stem cell-based embryo models (SCBEMs) have emerged as a promising tool in developmental biology, reproductive medicine, and regenerative science. Beyond their value for research, SCBEMs have also attracted considerable attention because they may address longstanding ethical and practical limitations associated with research using human embryos (Matthews and Moralí, 2020). In many jurisdictions, access to human embryos or embryonic stem cells remains limited, while research on human embryos is generally restricted by the internationally recognized 14-day limit that restricts culturing and research on human embryos to 14 days post-fertilization. SCBEMs, unlike embryos, are not formed through sperm–egg fusion and thus lack a defined “day 0” fertilization time point, a key reference point in the ethics of embryo research. These models thereby provide an experimentally tractable complementary model system that could circumvent these limitations in investigating early human development without relying on embryos (Nicolas et al, 2021). Consequently, SCBEMs have been proposed as an ethically acceptable alternative to human embryos that could help to advance our understanding of the mechanisms underlying early pregnancy loss, improve early pregnancy management, and aid the development of therapies for early developmental disorders (Brivanlou et al, 2021).

SCBEMs, unlike embryos, are not formed through sperm–egg fusion and thus lack a defined “day 0” fertilization time point, a key reference point in the ethics of embryo research.

Indeed, there is already a substantial body of literature on the ethical and regulatory challenges posed by this emerging technology. These regulatory attempts have been made not only internationally, led by organizations such as the International Society for Stem Cell Research (ISSCR) (Clark et al, 2021; International Society for Stem Cell Research, 2025) and the Nuffield Council on Bioethics (The Nuffield Council on Bioethics, 2024), but also by national legal frameworks such as that of the UK (Cambridge Reproduction, 2024) and the ones we shall discuss in this paper.

A crucial issue in these debates is the relationship between SCBEM research and the long-established 14-day limit. The ISSCR argues that SCBEMs could provide an ethically acceptable alternative to extending research on natural human embryos beyond 14 days, because they are not created through fertilization and are generally regarded as models mimicking embryos rather than actual embryos (ISSCR, 2025). At the same time, many scholars emphasize that this distinction may become progressively more difficult to sustain as SCBEMs acquire increasingly sophisticated developmental capacities that more closely replicate those of embryos (The Nuffield Council on Bioethics, 2024; Clark et al, 2025). Related discussions have also highlighted the need for conceptual clarity and procedural consistency in research (Holm, 2025), have reinterpreted the 2021 ISSCR framework with an eye to proportional oversight and international coherence (Clark et al, 2025), and characterized a “regulatory void,” urging the articulation of ethical imperatives (Vila-Pérez et al, 2025).

The ISSCR argues that SCBEMs could provide an ethically acceptable alternative to extending research on natural human embryos beyond 14 days…

Although scholars have compared and analyzed various regulatory approaches in this field across different countries (Matthews and Moralí, 2020), the regulatory situation in Asian countries remains unclear for most experts outside this region. This paper therefore discusses regulatory approaches in six Asian jurisdictions: China, Japan, Malaysia, Singapore, South Korea, and Taiwan. Although none of these countries has specific SCBEM legislation, their policies offer valuable insights for shaping regional governance and consensus.

## China

In April 2025, the Ministry of Science and Technology issued the *Ethical Guidelines for Human Organoid Research* that addresses ethical issues in research using human organoids, human–animal neural chimeras, and human SCBEMs. The *Guidelines* outline general principles applicable across these three areas—such as the role of research ethics committees and informed consent—while also setting out SCBEM-specific requirements, including criteria for terminating cultivation and an explicit prohibition on implanting human SCBEMs into a uterus. While most of these principles align broadly with international ethical consensus, scholars note that they are grounded in socialist and communitarian values, distinguishing them from Western, individually centered approaches (Zhou et al, 2025). Despite their designation as “guidelines,” they are regarded as binding norms, reflecting a state-led, top-down governance model (Zhou et al, 2025).

Beyond these newly promulgated *Guidelines*, China’s regulatory landscape for stem-cell research has been introduced in several scholarly articles (Peng et al, 2020; Peng et al, 2022). Since 2020, China has updated several major legal frameworks, including the Civil Code, the Criminal Law, the Biosecurity Law, and the Science and Technology Progress Law. Additional regulations are under consideration, such as a draft *Regulation on Safety Management of Biotechnology Research and Development* and the *Regulation on the Clinical Application of New Biomedical Technologies*.

Another foundational document is the 2003 *Ethical Guidelines for Human Embryonic Stem Cell Research*. Aligning with international trends, it sets a 14-day limit for embryo cultures and includes provisions such as: Prohibiting human cloning and human–animal chimera embryos; Restricting methods for obtaining human embryonic stem cells (hESCs); Banning embryo implantation into reproductive organs; Prohibiting the sale of gametes, embryos, or fetal cells; Requiring informed consent and privacy protection; and Mandating ethics review committees at research institutions.

Chinese law does not explicitly define an “embryo” (Matthews and Moralí, 2020; Peng et al, 2022), and regulation largely relies on guidelines rather than statutes. While guidelines may retain some binding force as mentioned above, they also afford flexibility in updating and revisions—a feature commentators view as an advantage for adapting to rapid scientific advances (The Nuffield Council on Bioethics 2024).

Chinese law does not explicitly define an “embryo” [..] and regulation largely relies on guidelines rather than statutes.

Ethical debates in China have been shaped by recurring tensions concerning the moral status of the embryo, which is generally understood as a form of biological life warranting respect but not full personhood (Peng et al, 2022). This view has supported a relatively permissive attitude toward the use and destruction of embryos in research when justified by potential medical benefits (Peng et al, 2020). The 14-day rule remains a key regulatory boundary, though ongoing discussions consider whether this limit should be extended to enable research on post-implantation development (Peng et al, 2022).

Further tensions arise from the relationship between rapid scientific innovation and regulatory adequacy. The He Jiankui incident—the CRISPR babies case—exposed weaknesses in existing ethical review systems and prompted a shift toward more systematic governance, strengthened through the Civil Code and Criminal Law (Peng et al, 2020; Peng et al, 2022; Xue and Shang, 2022). Additional areas of tension include the patentability of embryo-related technologies and persistent ambiguities in the legal definitions of embryos and related entities (Xue and Shang, 2022).

These debates are underpinned by a combination of Confucian gradualism, which attributes increasing moral status to developing human life, and strong state-driven commitments to scientific progress and economic development (Tang, 2025). Governance is primarily led by state institutions, with growing attention to “agile governance” and public deliberation to ensure that scientific advancement remains aligned with prevailing societal values and international expectations (Xue and Shang, 2022).

## Japan

In response to initiatives by the ISSCR, Japan has moved to formally regulate human SCBEM research. In November 2024, the Cabinet Office’s Expert Panel on Bioethics published an interim report recommending revisions to existing stem-cell research guidelines. Based on this report, revisions were made to the Guidelines on the Utilization of Human Embryonic Stem Cells and the Guidelines on the Research on Producing Germ Cells from Human iPS Cells or Human Tissue Stem Cells, which came into force in April 2026, formally extending their scope to research on human SCBEMs. 

Japan’s legal framework defines an embryo—under the Act on Regulation of Human Cloning Techniques—as a cell or a group of cells capable of developing into an individual through the process of development in utero. The expert panel considered whether SCBEMs are similar enough to natural human embryos to qualify as “specified embryos,” a legal category, which includes those derived by somatic cell nuclear transfer (SCNT), used to strictly regulate human cloning technologies. It concluded that SCBEMs, in their current form, differ significantly from both natural and cloned embryos, though future reconsiderations may be needed as science evolves.

Beyond legal classification, Japan’s revised guidelines established the following requirements. All research involving human SCBEMs must undergo ethics review and be reported to the Ministry of Education, Culture, Sports, Science, and Technology (MEXT): these notifications are published on MEXT’s website, functioning in practice as a national registration system. While these guidelines do not set a uniform upper limit on culture period of SCMEBs, researchers must define the minimum culture period needed to meet the research purposes, which is itself subject to ethics review. Research institutions are required to publish their research results and otherwise disclose information about the work. Where human iPSCs are used, the guidelines set out specific consent requirements. Finally, transferring SCBEMs into a human or animal uterus, and any attempt at further development using artificial gestation systems, is strictly prohibited, and institutional ethics committees must confirm that no project aims to produce a human individual.

Alongside the regulatory developments described above, ethical debates in Japan have been structured around a distinctive understanding of embryos as the “buds of human life,” which requires treating them with respect owing to their developmental potential. A key issue is therefore whether SCBEMs should be regarded as ethically equivalent to natural embryos or as fundamentally different entities. Current discussions tend to treat SCBEMs as cell aggregates distinct from embryos, while recognizing that this position may require revision if their developmental capacities change (Sawai et al, 2025). Moreover, Japan has adopted the international standard prohibiting the culture of human embryos beyond 14 days or the appearance of the primitive streak. While the ISSCR has relaxed this rule, Japanese discussions are ongoing regarding whether and how to adjust this limit to study early developmental processes (Sawai et al, 2025).

Beyond these ethical debates, the governance of SCBEM research in Japan faces a distinct set of structural challenges. Rather than arising primarily from overt moral polarization, these challenges concern the institutional structures and processes through which SCBEM research is governed. In particular, decision-making is largely concentrated in expert panels and government bodies, with limited public participation (Hyeon et al, 2023). At the same time, the regulatory system is often described as fragmented and complex. Guidelines have been dispersed across different documents and vary depending on research materials and contexts—for instance, different rules have governed the derivation, distribution, and use of human embryonic stem cells and iPSCs (Sawai et al, 2025). The 2026 revisions partly addressed this fragmentation: research using human iPSC–derived SCBEMs, which had previously fallen outside formal oversight when widely available cell lines were used, is now subject to ethics review and national-level notification requirements. 

## Malaysia

Malaysia does not currently have dedicated legislation governing human embryo and stem-cell research. Instead, oversight is primarily provided through guidelines supplemented by relevant legal and institutional governance mechanisms. As with many other jurisdictions, Malaysia has not yet adopted a regulatory framework specifically addressing SCBEMs.

As with many other jurisdictions, Malaysia has not yet adopted a regulatory framework specifically addressing SCBEMs.

The principal regulatory instrument is the *Guidelines on Stem Cell and Cell–Based Research and Therapy*, first issued by the Ministry of Health in 2006, updated in 2009, and revised in 2024. Although these Guidelines do not have the force of legislation, they provide the primary ethical and regulatory framework governing stem-cell research in Malaysia. Unlike Singapore or South Korea, which regulate embryo research through dedicated legislation, Malaysia relies principally on administrative guidance. Consequently, the Guidelines function as an important form of regulatory “soft law”: while they do not create statutory offenses, they are highly influential in institutional ethics review, research governance, and clinical practice.

The 2024 revision reaffirms several core principles established in earlier editions. They prohibit the creation of human embryos solely for research purposes, regardless of whether embryos are created through assisted reproductive technologies (ARTs) or SCNT. Research is permitted only on donated surplus embryos following in vitro fertilization (IVF), and only before 14 days of development or the appearance of the primitive streak. Although the Guidelines acknowledge the ISSCR’s 2021 recommendation that research beyond 14 days may be considered under specialized review on a case-by-case basis, Malaysia retains the traditional 14-day limit, reflecting a comparatively cautious regulatory approach.

Research involving hESCs and iPSCs is classified as “restrictive research” that requires approval from an Institutional Ethics Committee and compliance with enhanced governance and informed consent requirements. The Guidelines also prohibit reproductive cloning and specified forms of chimera research. Informed consent must be explicit, and renewed where necessary for specific research purposes, with no financial inducement permitted for embryo donation. In addition, researchers must ensure traceability of embryo-derived materials and maintain appropriate safeguards for long-term storage and confidentiality of stem-cell lines.

Still, regulatory challenges remain. Oversight by the Ministry of Health’s Medical Research and Ethics Committee (MREC) and the National Stem Cell Research and Ethics Subcommittee is primarily limited to public institutions, which may result in comparatively weaker oversight of the private sector. Furthermore, some forms of laboratory-based research that do not involve human participants may fall outside formal ethics review processes. Empirical studies also suggest that implementation challenges extend beyond the regulatory framework itself. Interviews with Malaysian stakeholders have demonstrated inconsistent awareness and interpretation of the Guidelines, indicating that effective governance depends not only on the existence of regulatory standards but also on institutional capacity, regulatory literacy, and consistent implementation (Bin Abdul Aziz et al, 2018).

Malaysia’s regulatory approach is closely shaped by its religious and cultural landscape. Although Malaysia is a multi-religious society, the development of national policies on embryo and stem-cell research has been substantially influenced by Islamic bioethical reasoning, particularly through guidance issued by the National Fatwa Council (Bin Abdul Aziz, 2025). At the same time, Malaysian policy also reflects broader efforts to accommodate the country’s religious diversity and maintain social legitimacy in biomedical governance.

The continued application of the 14-day rule is broadly consistent with the ethical position adopted by the National Fatwa Council. The resulting policy has been described as reflecting multi-religious consultation, leading to adoption of the 14-day limit rather than the classical Islamic view that ensoulment occurs later in fetal development. Ethical debate in Malaysia nevertheless remains centered on the moral status of the embryo and the permissibility of embryo destruction for research. A key distinction is drawn between surplus IVF embryos, which are generally regarded as ethically permissible for research, and embryos created specifically for research purposes, which are not (Sivaraman and Noor, 2017).

These debates reveal continuing tensions between religious doctrine, scientific advancement, and national research ambitions. While many Islamic scholars permit carefully regulated research on surplus embryos prior to ensoulment, Catholic perspectives regard human life as inviolable from the moment of fertilization and therefore reject embryo destruction (Bin Abdul Aziz, 2025). Other religious traditions adopt more context-dependent positions (Sivaraman and Noor, 2017). Consequently, policymakers must balance competing values, including the sanctity of life, public benefit, scientific progress, and social harmony, while also considering concerns that excessive regulatory restrictions may limit Malaysia’s competitiveness in regenerative medicine and biotechnology (Sivaraman and Noor, 2017; Bin Abdul Aziz et al, 2018; Nishakanthi, 2019).

Although the 2024 Guidelines regulate research involving embryos and embryonic stem cells, they do not expressly address SCBEMs or clarify whether these entities fall within existing provisions. Consequently, whether current restrictions on embryo creation, implantation, or developmental limits also apply to SCBEMs remains uncertain. Future governance will require policymakers to determine whether regulatory oversight should depend primarily on the origin of these entities, their developmental potential, or their functional characteristics.

## Singapore

Research on human embryos and hESCs is governed by the Human Biomedical Research Act (HBRA), which mandates strict informed consent and Institutional Review Board (IRB) oversight. With few exceptions, the HBRA generally applies to all human biomedical research and biobanking involving “individually-identifiable” biological materials (Labude, 2016). Thus, research using commercially available ESC lines—typically lacking donor-identifying information—is excluded. While commercial trading of human tissues is prohibited, this does not apply to commercially available cell lines.

Research involving human gametes and embryos is classified as “restricted” under the HBRA’s Fourth Schedule, and requires approval from both an IRB and the Director of Medical Services. The Healthcare Services Act, in its First Schedule, defines a human embryo as any live embryo with a human (or altered human) genome developing for fewer than 14 days since fertilization; the appearance of two pro-nuclei; or the initiation of its development by other means. Although the last part of this definition could in theory be interpreted as extending to certain forms of non-fertilization-based development, it remains unclear whether SCBEMs would fall within its scope. The legislation predates the development of SCBEMs and therefore could not have been drafted with them specifically in mind.

The HBRA prohibits transferring cloned embryos into human or animal uteruses but permits the culturing of embryos—regardless of whether they are created through fertilization or other processes—for up to 14 days. It remains uncertain how the 14-day limit applies to SCBEMs, which lack a clear developmental “Day 0.” The HBRA’s Third Schedule also bans research on human–animal combination (HAC) embryos beyond 14 days and any uterine implantation of HACs. Violations may incur substantial fines and/or imprisonment.

Complementing the HBRA, the Bioethics Advisory Committee (BAC)—established in 2000—issues non-legislative guidelines and conducts public consultations. While it has not yet addressed SCBEMs explicitly as such, the BAC plays a crucial role in balancing ethical responsibility with scientific and technological innovation in a broader context.

The debates on SCBEMs and human embryo research in Singapore are characterized by a structured approach to bioethics that balances national strategic goals in the biomedical sciences with the need to maintain social harmony in a multi-racial and multi-religious society. A central issue concerns the moral status of the embryo, with different views ranging from full moral status at fertilization to more gradualist interpretations. This diversity has led to the adoption of intermediate regulatory positions that permit research under defined conditions while maintaining ethical safeguards.

The BAC adopts an inclusive model that actively engages faith communities and the public in dialogue before making recommendations, ensuring that public policy is based on a “considered weighting and balancing” of diverse views. Its discussions are grounded in established principles, including respect for persons, solidarity, justice, proportionality, and sustainability. Within this framework, Chin et al (2025) have argued that SCBEMs raise questions about whether they should be treated as equivalent to embryos and, more specifically, whether their application in human reproduction should be prohibited on the grounds that they may constitute human cloning because they may be genetically identical to the cell from which they were derived.

Relatedly, a pervasive social value potentially reinforcing this stance is the primacy of “blood ties” and “genetic connection” in family formation, reflecting traditional Confucian norms that influence both sociocultural attitudes and legal rulings in family courts. Ultimately, while Singapore’s regulatory framework is robust, the exact legal classification of SCBEMs remains unclear, as the “other initiation methods” clause has never been tested by the courts. Although a government authority could interpret the provision as extending to some SCBEMs, its legal scope would ultimately fall to the courts to determine. In practice, however, such judicial review is unlikely, making regulatory clarification important to avoid inconsistent or arbitrary interpretation.

Ultimately, while Singapore’s regulatory framework is robust, the exact legal classification of SCBEMs remains unclear, as the “other initiation methods” clause has never been tested by the courts.

## South Korea

South Korea currently has no specific regulations on SCBEMs, but they are generally governed under the Bioethics and Safety Act (BSA). Originally enacted after public debate on the birth of Dolly the sheep (Jung, 2010), the BSA regulates human subjects research, the use of human biological materials, and the handling of embryos. It also defines key terms like embryo and embryonic stem-cell line: an embryo is defined as a fertilized human egg or a group of dividing cells from the time of fertilization until the point at which all organs are formed embryologically. The law prohibits human cloning and xenotransplantation.

Only designated medical institutions may collect, preserve, and fertilize gametes for IVF, exclusively for pregnancy. Creating embryos for any other purposes is banned, along with practices like sex-selective fertilization and using gametes from minors or the deceased. Embryos may be stored up to five years, after which surplus embryos may be used for research only before the primitive streak forms—around day 14 post-fertilization. Research is allowed for developing infertility treatments or contraceptives; research on rare or incurable diseases, as defined by Presidential Decree; and other purposes approved by Presidential Decree following deliberation by the National Bioethics Committee. Researchers must register with the Minister of Health and Welfare and submit IRB-approved proposals. Somatic-cell cloning embryos may only be used to develop treatments for rare or incurable diseases under similar conditions.

ESC lines must be registered before use or distribution. Their use is limited to research on diagnosis, prevention, or treatment of diseases; basic studies on stem-cell properties and differentiation; and other research purposes approved by Presidential Decree. All such research requires IRB review and institutional approval.

Under the BSA’s Article 47, gene therapy is permitted only for life-threatening or serious conditions—such as genetic disorders, cancer, and acquired immune deficiency syndrome (AIDS)—where there is no existing effective treatment, or where gene therapy is expected to be significantly superior to existing therapies. However, genetic interventions on embryos, gametes, or fetuses for therapeutic purposes are strictly prohibited. Additionally, the Act on the Safety of and Support for Advanced Regenerative Medicine and Advanced Biological Products governs clinical trials and safety management of cell-, gene- and tissue-based therapies, aiming to ensure the safe development of regenerative treatments.

A central issue in South Korea concerns the moral status of the embryo, with differing views on whether it should be regarded as a human being from fertilization or at later developmental stages. Regarding the classification of SCBEMs, current legislation defines an embryo based on fertilization. Therefore, SCBEMs, which are generated from stem cells without fertilization, fall outside the regulatory framework governing embryos. Nevertheless, they remain subject to the regulations governing research involving human biological materials or hESC lines.

The ethical landscape has also been partly influenced by the Hwang Woo-Suk scandal and the ensuing public dispute, which exposed serious ethical violations in egg procurement and research practices and prompted concerns about informed consent, health risks, and the commercialization of the human body (Jung, 2010). This legacy of public distrust has fueled sharp confrontations between the scientific community and civil society, with critiques often coming from progressive groups that view the “rush” toward embryo research as a threat to democracy and social justice (Kim, 2014). Consequently, while SCBEMs currently appear to fall outside existing legal constraints on embryos under South Korea’s fertilization-based definition of embryo, any attempt to exploit this regulatory loophole would likely trigger social and ethical backlash, unless preemptively addressed by policymakers.

…while SCBEMs currently appear to fall outside existing legal constraints on embryos under South Korea’s fertilization-based definition of embryo, any attempt to exploit this regulatory loophole would likely trigger social and ethical backlash…

## Taiwan

In June 2024, Taiwan enacted the Regenerative Medicine Act, which formalizes and expands earlier guidelines, including the *Guidelines for Ethical Policy on Human Embryo and Embryonic Stem Cell Research*. These guidelines prohibit creating human embryos for research using donor gametes via assisted reproductive technologies (ART). Under the Assisted Reproduction Act, a “human embryo” is defined as a fertilized oocyte that has divided for less than eight weeks. Permissible sources of embryos or ESCs include tissues from natural miscarriages (donated without compensation); tissues from abortions under the Reproductive Health Act; surplus ART embryos; and embryos or tissues produced via SCNT.

Aside from this legislation, key regulations include the *Notice on the Collection and Use of Human Specimens for Research Use*, with which all research use of embryos, ESCs, or reproductive cells must comply; and the *Regulations on the Import and Export of Human Organs and Tissue Cells*, which prohibit exporting hESCs or ESC lines from Taiwan without approval from the Ministry of Health and Welfare. The 14-day rule is mentioned in several policy documents like the *Code of Ethics for Embryonic Stem Cell Research or the Guidelines for Ethical Policy on Human Embryo and Embryonic Stem Cell Research* (Tsai, 2020).

As of now, there is no active discussion on SCBEMs in Taiwan. According to an expert consultation by the Taiwan Science Media Center in June 2023, human SCBEM research is virtually absent in Taiwan, especially compared to vibrant organoid studies. They conclude that, without due public engagement and regulatory development, such research may remain constrained by ethical concerns and the 14-day rule.

Similar to South Korea, an issue potentially raised in Taiwan’s governance of SCBEM research is its fertilization-based definition of embryos, which does not technically apply to SCBEMs. Beyond that, the debates regarding SCBEMs, hESC research, and gene editing in Taiwan are shaped by two intersecting forces: a strong national drive toward biotechnological innovation and a gradualist view of human life rooted in Confucian thought. On the ethical side, Taiwanese society generally holds that an embryo’s moral status increases as it develops rather than being fixed at fertilization. Accordingly, the prevailing perspective treats embryos as “quasi-humans” who are not yet legal subjects of rights but deserve special respect, though some religious views equate their destruction with killing (Hsiao, 2010). On the policy side, Taiwan views biotechnology as a critical pillar of its economy—reflected in initiatives such as the “Two Trillion Two Stars” industries that favor research and development to maintain national competitiveness. Tensions arise at the intersection of these forces, particularly in relation to commercialization, patentability, and ambiguity in the regulatory frameworks governing embryo use (Hsiao, 2010). Since Taiwan’s Assisted Reproduction Act explicitly defines an embryo as a “fertilized oocyte,” SCBEMs are technically excluded from current embryo regulations, and addressing this regulatory gap is needed to prevent unguided SCBEM research from crossing established ethical boundaries.

Since Taiwan’s Assisted Reproduction Act explicitly defines an embryo as a “fertilized oocyte,” SCBEMs are technically excluded from current embryo regulations…

## Discussion

Table 1 summarizes the regulatory status of SCBEM research in six Asian countries, highlighting key instruments and whether SCBEMs are explicitly addressed. Despite legal definitions of an embryo, SCBEMs have historically fallen into a regulatory gray zone in many of these jurisdictions, although recent developments in Japan illustrate an effort to address this gap explicitly. Japan’s legal definition of an embryo is based on the capacity of a cell or group of cells to develop into an individual through development in utero. Under the current regulatory interpretation, SCBEMs in their present form are not treated as embryos or specified embryos under this framework; instead, human SCBEM research is explicitly governed by revised stem-cell research guidelines that came into force in April 2026. By contrast, South Korea’s statutory definition of an embryo is tied to fertilization, which does not straightforwardly encompass SCBEMs because they are not created through sperm–egg fusion. China and Malaysia lack statutory definitions altogether, which leaves SCBEMs entirely subject to ad hoc institutional interpretations. Conversely, Singapore’s mention of “other initiation methods” offers a possible, albeit ambiguous regulatory hook that may be too imprecise to capture a technology that did not exist at the time the definition was written. Consequently, these differences illustrate that SCBEMs do not fit neatly within conventional embryo-based regulatory categories and underscore the need for jurisdictions to clarify explicitly how existing or new regulatory frameworks apply to SCBEM research.

**Table 1:** Comparative summary of regulatory approaches to SCBEM research in Asian countries

	Key regulatory instruments	Legal definition of embryo	SCBEMs explicitly addressed?	14-Day rule
**China**	Ethical Guidelines for Human Organoid Research (2025); Ethical Guidelines for Human Embryonic Stem Cell Research (2003); Biosecurity Law (2021)	No statutory definition	Yes (2025 Guidelines)	Guidelines-based (14 days/primitive streak; established in 2003 hESC Guidelines)
**Japan**	Act on Regulation of Human Cloning Techniques (2000); Interim Report of the Expert Panel on Bioethics (2024); Guidelines on the Utilization of Human Embryonic Stem Cells (rev. 2026); Guidelines on the Research on Producing Germ Cells from Human iPS Cells or Human Tissue Stem Cells (rev. 2026)	A cell (other than a sperm or egg) or a cell group capable of developing into an individual through the process of development in utero, and is at a stage prior to placental formation (ARHCT)	Yes (revised guidelines in force since April 2026)	Guidelines-based for embryos (14 days/primitive streak); for SCBEMs, no maximum culture period (subject to scientific necessity and ethics review)
**Malaysia**	Guidelines on Stem Cell and Cell-Based Research and Therapy (2006, rev. 2024)	(No statutory definition)	No. Potentially falling under broader stem cell guidelines	Guidelines-based (14 days/primitive streak; affirmed in 2024 Guidelines based on Fatwa Council ruling)
**Singapore**	Human Biomedical Research Act; Human Cloning and Other Prohibited Practices Act; Healthcare Services Act; Bioethics Advisory Committee guidelines	Live embryo with a human (or altered human) genome developing for fewer than 14 days since (a) fertilization, (b) the appearance of 2 pro-nuclei, or (c) other initiation methods (HSA)	No. Potentially falling under the “other initiation methods” clause	Statutory (14 days; governed under HBRA and HCPA)
**South Korea**	Bioethics and Safety Act (2005, rev. 2025); Act on Regenerative Medicine (last amended 2024)	A fertilized human egg or a group of divided cells from the time of fertilization until the point at which all organs are formed embryologically (BSA)	No. Potentially falling under the BSA’s regulations on research involving human biological materials or the use of hESC lines	Implicit (Statutory) (Primitive streak formation, approx. 14 days; regulated under BSA)
**Taiwan**	Regenerative Medicine Act (2024); Assisted Reproduction Act (2007, rev. 2018); Guidelines for Ethical Policy on Human Embryo and ESC Research (2007)	A fertilized oocyte that has divided for less than eight weeks (ARA)	No. Excluded from ARA’s oversight due to its fertilization-based definition of embryos	Guidelines-based (14 days; referenced in ESC Code of Ethics and hESC Guidelines)

*ARHCT* Act on Regulation of Human Cloning Techniques, *HSA* Healthcare Services Act, *BSA* Bioethics and Safety Act, *ARA* Assisted Reproduction Act, *HBRA* Human Biomedical Research Act, *HCPA* Human Cloning and Other Prohibited Practices Act.

Despite legal definitions of an embryo, SCBEMs largely fall into a regulatory gray zone in all six jurisdictions.

As of now, China and Japan are the only countries in the region to have formally begun regulating SCBEMs. Elsewhere, such research likely falls under broader embryo or stem-cell regulations until more specific guidance is developed. Understanding these varying approaches is crucial for promoting international dialogue. As SCBEM research grows, countries will increasingly need to clarify their legal and ethical positions.

Across all countries examined, a shared set of core ethical issues can be identified, including the moral status of the embryo and the role and limits of the 14-day rule. At the same time, these shared issues give rise to different types of conflicts depending on national contexts. In several jurisdictions, tensions are observed between scientific innovation and ethical or social constraints. In China and South Korea, these tensions have been particularly visible amid rapid scientific development and high-profile controversies, leading to a subsequent strengthening of regulatory oversight. In contrast, Japan’s debates are characterized more by procedural and institutional tensions, such as the concentration of decision-making within expert bodies and the complexity of overlapping guidelines.

In pluralistic societies, such as Malaysia and Singapore, conflicts often reflect diverse religious and moral perspectives, particularly regarding when human life begins and the permissibility of embryo research. These differences are managed through efforts to establish intermediate or compromise positions that allow research while maintaining social legitimacy. Taiwan presents a further variation, where tensions arise between economic ambitions in biotechnology and concerns about ethical boundaries and regulatory clarity. Gradualist views of embryonic moral status, often influenced by Confucian traditions, are evident in China, Japan, and Taiwan, while Malaysia’s debates are shaped by multi-religious ethical perspectives.

An important implication of these regulatory differences concerns the 14-day rule. Since SCBEMs are not produced by fertilization, they often fall outside the existing statutory definitions of human embryo, and current embryo legislation may not automatically apply to SCBEM research. This gap creates two possibilities. On the one hand, SCBEMs may enable researchers to investigate developmental events that are ethically or practically difficult to study using human embryos. On the other hand, if increasingly sophisticated SCBEMs acquire developmental capacities approaching those of embryos, such fertilization-based legal definitions may create regulatory inconsistencies and ethical loopholes that may need revision.

This urgency is further compounded by the structural limitations of existing oversight mechanisms. In jurisdictions like Malaysia, where guidelines apply primarily to public institutions, this regulatory gray zone could easily transform into a critical enforcement gap. In the rapidly globalizing market for biotechnology, such domestic discrepancies risk facilitating premature commercialization or fueling an unregulated “stem-cell tourism,” where private clinics or commercial entities exploit legal loopholes to offer unproven stem cell-based interventions or conduct commercial research services, such as toxicological screening on unvetted embryo models, without rigorous ethical review. Therefore, regional governance must not only clarify the definition of embryos but also ensure that compliance is legally mandated across both public and private entities.

Besides the question of whether the 14-day rule should be revised, the current international debate also concerns whether the relevant regulatory oversight should continue to depend primarily on an entity’s origin or on its biological properties and developmental potential (The Nuffield Council on Bioethics, 2024; Aach et al, 2017; McCully, 2021). As the international bioethics community contemplates a shift from origin-based frameworks to ones based on the biological or developmental properties, East Asian jurisdictions—particularly China, Japan, and Taiwan—possess a distinctive conceptual advantage. Grounded in Confucian gradualism, these societies traditionally view the moral status of developing human life not as a binary state fixed at fertilization, but as a relational and gradual accretion of moral worth that increases alongside biological development. This culturally embedded gradualism shares a conceptual affinity with a property-based regulatory approach, which scales the level of ethical oversight to the actual biological capacities—such as sentience or neural complexity—exhibited by an embryo model at any given stage.

Conversely, in traditions characterized by a non-gradualist, origin-centered ontology—where moral status is absolute from fertilization or ensoulment—transitioning to a property-based model of governance often triggers polarization. Critically, the East Asian gradualist approach should not be conflated with a permissive “slippery slope” that inevitably erodes ethical boundaries. Far from licensing unregulated scientific exploitation, Confucian gradualism represents a robust normative framework that imposes increasingly stringent relational responsibilities and ethical duties of respect as a biological entity acquires greater developmental complexity. Thus, the pragmatic and progressive regulatory steps observed in East Asia suggest that the region’s philosophical heritage is not merely a local cultural variance, but a valuable theoretical resource capable of facilitating a smoother, more coherent transition toward dynamic, property-based global governance for frontier biotechnology.

… Confucian gradualism represents a robust normative framework that imposes increasingly stringent relational responsibilities and ethical duties of respect as a biological entity acquires greater developmental complexity.

## Supplementary information

Peer Review File
