# Supplementary material for: What counts as an embryo? The regulatory status of stem cell-based embryo models in Asia
Source: EMBO Rep. 2026 Aug 19;27(18):5178–86. doi: 10.1038/s44319-026-00903-4 (PMC13601569; doi:10.1038/s44319-026-00903-4)
Supplement: Supplementary file 1 [file 44319_2026_903_MOESM1_ESM.pdf]

EMBO reports

What Counts as an Embryo?: Regulatory status of stem cell-based embryo models in Asia

Tsutomu Sawai, Shu Ishida, Chie Kobayashi, Yasuna Ando Murase, Daniel Tsai, Ji Hyun Yang, Ilhak Lee, Tamra Lysaght, Mohammad Firdaus Bin Abdul Aziz, and Julian Savulescu

Corresponding authors: Tsutomu Sawai (tstmsw@hiroshima-u.ac.jp) , Julian Savulescu (jsavules@nus.edu.sg)

Review Timeline:

Submission Date:	3rd Aug 26
Accepted:	6th Aug 26

Editor: Holger Breithaupt

Transaction Report:

This article was editorially reviewed.
